# Paracetamol in pregnancy: Navigating clinical uncertainty and avoiding the communication pitfalls of the “measles, mumps, and rubella” - autism controversy: A narrative review

**DOI:** 10.17179/excli2026-9237

**Published:** 2026-04-07

**Authors:** Helmi Ben Saad, Chamseddine Barki, Ismail Dergaa, Wissem Dhahbi, Halil Ibrahim Ceylan, Nasr Chalghaf, Abdelfatteh EL Omri, Hanene Boussi Rahmouni

**Affiliations:** 1 University of Sousse, Faculty of Medicine 'Ibn el Jazzar' of Sousse, Farhat Hached University Hospital, Research Laboratory LR12SP09 'Heart Failure', Sousse, Tunisia; 2 Department of Physiology and Functional Explorations, Farhat Hached University Hospital, Sousse, Tunisia; 3 University Central Group, UPSAT-Sousse, CRCI, Tunis, Tunisia; 4 Research Laboratory of Biophysics and Medical Technologies, Higher Institute of Medical Technologies of Tunis, University of Tunis El Manar, Tunis 1006, Tunisia; 5 High Institute of Sport and Physical Education of Ksar Said, University of Manouba, Manouba 2010, Tunisia; 6 Physical Activity Research Unit, Sport and Health (UR18JS01), National Observatory of Sports, Tunis 1003, Tunisia; 7 High Institute of Sport and Physical Education of Kef, University of Jendouba, Kef 7100, Tunisia; 8 Training Department, Police College, Police Academy, Doha 7157, Qatar; 9 Physical Education of Sports Teaching Department, Faculty of Sports Sciences, Atatürk University, Erzurum 25240, Türkiye; 10 High Institute of Sport and Physical Education of Gafsa, University of Gafsa, Gafsa 2100, Tunisia; 11 Surgical Research Section, Department of Surgery, Hamad Medical Corporation, Doha, Qatar

**Keywords:** acetaminophen, autism spectrum disorder, evidence-based medicine, health communication, narrative review, neurodevelopmental disorders, paracetamol, pregnancy, risk communication, shared decision-making

## Abstract

On September 22, 2025, the United States government announced that the Food and Drug Administration (FDA) would modify paracetamol (acetaminophen) labelling to warn of possible associations with autism, advising pregnant individuals to avoid the medication. This contradicts professional medical consensus and high-quality evidence, replicating communication failures of the 1998 MMR-autism controversy that caused vaccine hesitancy, disease outbreaks, and trust erosion. This narrative review synthesized epidemiological evidence on paracetamol safety in pregnancy, analyzed the September 2025 announcement through the measles, mumps, and rubella (MMR)-autism crisis lens, and proposed an evidence-based communication framework. We searched *PubMed*, *Embase*, *Web of Science*, and *Google Scholar*, supplemented with governmental statements, professional responses, and media analysis. The two highest-quality sibling-control studies (Swedish: 2.5 million; Japanese: 200,000 children) reported no causal associations between prenatal paracetamol exposure and neurodevelopmental outcomes after controlling genetic and familial confounding. Conversely, untreated maternal fever and pain carry established risks including neural tube defects, preterm birth, and maternal morbidity. The governmental announcement employed inflammatory categorical warnings contradicting FDA's nuanced advisory and scientific consensus. Professional organizations immediately issued strong rebuttals. This replicates MMR failures: governmental statements contradicting evidence, false media balance, and public confusion. The September 2025 announcement represents failure to apply MMR lessons. Healthcare providers must employ evidence-based shared decision-making emphasizing sibling-controlled studies show no causal relationship while untreated conditions carry established harms. The Precautionary Communication Principle provides framework for transparent uncertainty discussion without disproportionate alarm or undermining evidence-based medicine trust.

See also the graphical abstract(Fig. 1).

## Abbreviations

ACOG: American College of Obstetricians and Gynecologists

ADHD: Attention-Deficit/Hyperactivity Disorder

CB1: Cannabinoid Receptor Type 1

CDC: Centers for Disease Control and Prevention

CI: Confidence Interval

DNA: Deoxyribonucleic Acid

EMA: European Medicines Agency

FACT: Evidence Suggests Link between Acetaminophen, Autism

FDA: Food and Drug Administration

HHS: Health and Human Services

HR: Hazard Ratio

MMR: Measles, Mumps, and Rubella

NAPQI: N‑Acetyl‑P‑Benzoquinone Imine

OS: Oxidative Stress

RCOG: Royal College of Obstetricians and Gynaecologists

SMFM: Society for Maternal-Fetal Medicine

## Introduction

On September 22, 2025, a United States governmental announcement indicated that the Food and Drug Administration (FDA) would modify labelling for paracetamol (acetaminophen, branded as Tylenol in the United States) to warn of possible associations with autism spectrum disorder when used during pregnancy (Horton, 2025[53]; Pearson and Ledford, 2025[84]). The announcement included categorical warnings advising pregnant individuals to avoid paracetamol during pregnancy (Horton, 2025[53]; Pearson and Ledford, 2025[84]). The White House released a document titled “FACT: Evidence Suggests Link between Acetaminophen, Autism” (Lavery, 2025[64]), while Health and Human Services (HHS) Secretary Robert F. Kennedy Jr. stated the FDA would update safety labelling accordingly (Horton, 2025[53]; Lavery, 2025[64]; Pearson and Ledford, 2025[84]). This governmental announcement contradicts the consensus of major professional medical organizations (Louwen et al., 2025[73]; EMA, 2025[36]; Szubert et al., 2025[98]) and misrepresents the highest-quality scientific evidence (Ahlqvist et al., 2024[3]; Okubo et al., 2026[82]), replicating communication failures that characterized the 1998 measles, mumps, and rubella (MMR)-vaccine-autism controversy (Deer, 2011[27]; Godlee et al., 2011[41]; Wakefield et al., 1998[105]). The American College of Obstetricians and Gynecologists (ACOG) immediately affirmed paracetamol safety (Szubert et al., 2025[98]), while the Society for Maternal-Fetal Medicine (SMFM) issued similar rebuttals (Louwen et al., 2025[73]). International scientific experts interviewed by Nature emphasized, “there is no robust evidence or convincing studies to suggest there is any causal relationship” (Pearson and Ledford, 2025[84]). The FDA itself issued a more measured advisory: “It is important to note that while an association between acetaminophen and neurological conditions has been described in many studies, a causal relationship has not been established, and there are contrary studies in the scientific literature” (FDA, 2025[39]). This divergence emphasizes the political rather than scientific nature of this controversy (Horton, 2025[53]; Pearson and Ledford, 2025[84]; FDA, 2025[39]).

The September 2025 events demonstrate what occurs when historical lessons about health communication failures are ignored (Godlee et al., 2011[41]; Leask and Chapman, 1998[66]; Wakefield et al., 1998[105]). The MMR vaccine-autism controversy, which began with Andrew Wakefield's fraudulent 1998 Lancet paper, caused measles outbreaks, preventable deaths, and lasting erosion of vaccine confidence (Godlee et al., 2011[41]; Jansen et al., 2003[56]; Salmon et al., 2006[93]; Wakefield et al., 1998[105]). Analysis identified specific communication failures: premature causal claims despite weak evidence, governmental amplification of fringe positions, false balance in coverage, and inadequate attention to methodological quality (Balog-Way and McComas, 2025[8]; Leask et al., 2010[65]; Leask and Chapman, 1998[66]). The September 2025 paracetamol announcement exhibits identical failures (Horton, 2025[53]; Lavery, 2025[64]; Pearson and Ledford, 2025[84]). Paracetamol has been widely used during pregnancy for decades (Bauer et al., 2021[10]; Werler et al., 2005[106]), with governmental agencies including the FDA and European Medicines Agency (EMA) considering it the safest option for pain and fever relief when used as directed (Bauer et al., 2021[10]; Luxey et al., 2025[74]). More than 50 % of pregnant women worldwide use paracetamol during gestation (Chen et al., 2019[21]; Werler et al., 2005[106]). Other analgesics including non-steroidal anti-inflammatory drugs carry known risks of miscarriage, birth defects, and adverse fetal outcomes after 20 weeks' gestation (Antonucci et al., 2012[5]; Daniel et al., 2014[24]), while opioids present risks of neonatal abstinence syndrome (Kocherlakota, 2014[61]), making paracetamol the sole recommended antipyretic and analgesic for pregnant individuals (Louwen et al., 2025[73]; Luxey et al., 2025[74]; Szubert et al., 2025[98]).

Concerns about paracetamol neurodevelopmental effects emerged from observational epidemiological studies published between 2014 and 2021 (Bauer et al., 2018[9]; Liew et al., 2014[70]; Ystrom et al., 2017[110]), reporting associations between prenatal paracetamol exposure and increased risks of autism, Attention-Deficit/Hyperactivity Disorder (ADHD), and behavioral problems (Bauer et al., 2018[9]; Liew et al., 2014[70]; Ystrom et al., 2017[110]). A September 2021 consensus statement by 91 scientists called for precautionary action (Bauer et al., 2021[10]), generating substantial controversy (Nilsen et al., 2023[81]; O'Sullivan et al., 2022[83]). Critical methodological limitations plague such research (Hernán et al., 2004[49]; Tyrrell et al., 2012[102]; VanderWeele and Ding, 2017[104]). Most studies rely on maternal recall, introducing measurement error (VanderWeele and Ding, 2017[104]). More critically, few adequately control for confounding by indication: women using paracetamol during pregnancy do so because of fever, pain, or other conditions that may themselves affect neurodevelopmental outcomes (Hernán et al., 2004[49]; VanderWeele and Ding, 2017[104]). Genetic confounding represents another threat (D'Onofrio et al., 2013[31]; Tyrrell et al., 2012[102]).

Two landmark studies using sibling-control designs fundamentally challenged causal interpretations (Ahlqvist et al., 2024[3]; Okubo et al., 2026[82]). A 2024 Swedish study examined 2,480,797 children born between 1995 and 2019 (Ahlqvist et al., 2024[3]). While conventional analyses reported modest associations between paracetamol exposure and autism/ADHD, sibling-controlled analyses highlighted no associations (Ahlqvist et al., 2024[3]), indicating that unmeasured familial and genetic factors explain apparent associations (Ahlqvist et al., 2024[3]; D'Onofrio et al., 2013[31]). A 2025 Japanese study of over 200,000 children reached identical conclusions (Okubo et al., 2026[82]). These represent the highest-quality evidence available, yet the September 2025 governmental announcement minimized or ignored these findings (Horton, 2025[53]; Lavery, 2025[64]; Pearson and Ledford, 2025[84]).

Risks of untreated maternal fever and pain remain well-established (Bjelland et al., 2003[12]; Dreier et al., 2014[32]; Polifka and Friedman, 2002[87]). Maternal fever, particularly during the first trimester, substantially increases risks of neural tube defects, congenital heart defects, and oral clefts (Dreier et al., 2014[32]; Polifka and Friedman, 2002[87]). Untreated pain contributes to maternal depression, anxiety, and complications (Bjelland et al., 2003[12]; Gutke et al., 2007[46]). The Centers for Disease Control and Prevention (CDC) classify fever exceeding 38 °C (i.e., 100.4 °F) during pregnancy as an “urgent maternal warning sign” requiring immediate treatment (Wisner, 2021[107]). The September 2025 announcement also occurred alongside renewed governmental attention to vaccine-autism links (Gulati et al., 2025[45]; Horton, 2025[53]; Pearson and Ledford, 2025[84]). Despite decades of research refuting any association between vaccines and autism (Hviid et al., 2019[55]; Madsen et al., 2002[75]; Taylor et al., 2014[101]), governmental authorities announced in March 2025 that the CDC would launch a study examining potential vaccine-autism connections (Gulati et al., 2025[45]), raising concerns that the paracetamol announcement represents policy decisions disconnected from evidence-based medicine (Horton, 2025[53]; Pearson and Ledford, 2025[84]). This controversy highlighted concerns about over-the-counter availability of paracetamol during pregnancy. While paracetamol remains the preferred analgesic-antipyretic when medically indicated, the absence of mandatory medical consultation before purchase creates potential for inappropriate use patterns (ACOG, 2025[1]; ENTIS, 2021[37]; UKTIS, 2025[103]). Professional organizations recommend that pregnant individuals consult healthcare providers before using paracetamol (ACOG, 2025[1]; ENTIS, 2021[37]; UKTIS, 2025[103]). This approach allows proper assessment of indication, appropriate dosing, shortest effective duration, and gestational timing.

This narrative review addresses several urgent questions. What does the highest-quality evidence reveal about associations between prenatal paracetamol exposure and neurodevelopmental outcomes? How do the September 2025 governmental statements compare to scientific evidence and professional medical consensus? What specific parallels exist between the MMR-autism and paracetamol-autism communication failures? What practical framework can guide clinicians in shared decision-making with concerned patients? What broader implications does this episode hold for evidence-based medicine and public trust in health authorities?

## Materials and Methods

### Review design and rationale

This narrative review employed an integrative analytical framework combining systematic evidence synthesis with real-time policy analysis (Green et al., 2006[42]; Petticrew and Roberts, 2008[85]). We synthesized epidemiological evidence on paracetamol safety. We analyzed September 2025 governmental statements and responses. We compared current events to the historical MMR-autism controversy (Green et al., 2006[42]). This approach permits examination of how scientific evidence translates, or fails to translate, into public health policy and communication (Greenhalgh et al., 2018[43]; Petticrew and Roberts, 2008[85]). Given the rapidly evolving nature of the September 2025 events and the explicit aim of integrating real-time policy analysis with historical comparison, a narrative review was deemed more appropriate than a full systematic review with prospective registration. This allowed timely synthesis of emerging governmental statements, professional responses, and media coverage alongside established epidemiological evidence.

### Literature search strategy

Comprehensive searches were conducted across *PubMed*, *Embase*, *Web of Science*, and *Google Scholar* through September 25, 2025. For paracetamol evidence: (paracetamol OR acetaminophen OR APAP) AND (pregnan* OR prenatal) AND (autism OR ADHD OR neurodevelop*). For communication literature: (“risk communication” OR “health communication”) AND (uncertain* OR “precautionary principle”) AND (vaccine* OR MMR). Searches included Medical Subject Headings terms and truncation symbols (Bramer et al., 2017[15]).

Supplementary sources included: (1) Official governmental statements from the White House, FDA, and the Department of HHS; (2) Professional organization responses from the ACOG, the SMFM, the Royal College of Obstetricians and Gynaecologists (RCOG), and the EMA; (3) Scientific expert commentary in Nature, BMJ, and other outlets; (4) Media analysis including fact-checking organizations; and (5) Forward and backward citation tracking (Atkins et al., 2004[6]).

### Inclusion criteria and selection

We included: (1) Original epidemiological research on prenatal paracetamol exposure and neurodevelopmental outcomes, prioritizing sibling-controlled studies; (2) Systematic reviews and meta-analyses; (3) Official governmental and organizational statements from September 2025; (4) Expert commentary and analysis; (5) Historical research on the MMR-autism controversy; and (6) Theoretical work on risk communication and shared decision-making (Green et al., 2006[42]; Petticrew and Roberts, 2008[85]).

### Analytical framework

Analysis integrated several frameworks (Greenhalgh et al., 2018[43]): (1) Epidemiological critical appraisal emphasizing study design hierarchy, confounding control, and causal inference (Guyatt et al., 2008[47]; Schünemann et al., 2019[95]); (2) Content analysis of governmental statements, professional responses, and media coverage (Eysenbach, 2009[38]); (3) Comparative historical analysis of MMR and paracetamol controversies (Leask et al., 2010[65]; Leask and Chapman, 1998[66]); (4) Communication theory examining trust, credibility, and false balance (Covello et al., 2001[23]; Slovic, 1999[97]); and (5) Bioethics frameworks addressing precaution, autonomy, and evidence-based practice (Beauchamp and Childress, 2019[11]; Kass, 2001[59]).

## Results

### Literature search and included studies

Searches yielded 2,134 potentially relevant records. After removing duplicates and screening, 78 publications were included: 19 original epidemiological studies, 6 systematic reviews, 10 official statements from September 2025, 15 historical MMR-autism publications, 16 communication theory articles, eight shared decision-making studies, and four bioethics papers. Table 1(Tab. 1) presents characteristics of included literature.

### The September 2025 governmental announcement: Content analysis

#### White House statements

The September 22, 2025 governmental announcement contained categorical warnings contradicting scientific evidence (Horton, 2025[53]; Pearson and Ledford, 2025[84]). Official statements from this announcement included categorical warnings advising against paracetamol use and unverified claims about international autism prevalence (Horton, 2025[53]). These statements employed several problematic rhetorical strategies (Horton, 2025[53]; Pearson and Ledford, 2025[84]): (1) Categorical imperatives rather than nuanced risk-benefit discussion; (2) Inflammatory language; (3) Unverified anecdotes about Cuba with no supporting evidence; (4) No acknowledgment of scientific uncertainty or conflicting evidence; (5) No discussion of risks from untreated fever and pain. The White House FACT sheet similarly presented associations as established fact (Lavery, 2025[64]). The document cited select observational studies showing associations while minimizing the highest-quality sibling-controlled studies showing null findings (Lavery, 2025[64]).

#### FDA advisory: Contrast with White House messaging

The FDA's September 22, 2025 advisory presented markedly different messaging (FDA, 2025[39]). The agency stated: “It is important to note that while an association between acetaminophen and neurological conditions has been described in many studies, a causal relationship has not been established, and there are contrary studies in the scientific literature” (FDA, 2025[39]). The latter statement: (1) Distinguished association from causation; (2) Acknowledged contrary evidence; (3) Noted scientific uncertainty; (4) Used measured language. The divergence between White House and FDA messaging reveals tensions between political and scientific communication (Horton, 2025[53]; Pearson and Ledford, 2025[84]; FDA, 2025[39]).

#### Professional medical organization responses

Major medical organizations responded swiftly with statements contradicting the governmental warnings (BJOG, 2025[13]; Louwen et al., 2025[73]; EMA, 2025[36]; Szubert et al., 2025[98]). ACOG issued a statement on September 22, 2025 (Szubert et al., 2025[98]): “The data from numerous studies have shown that acetaminophen plays an important, and safe, role in the well-being of pregnant women… The two highest-quality studies on this subject, one of which was published in JAMA last year, found no significant associations between use of acetaminophen during pregnancy and children's risk of autism, ADHD, or intellectual disability”. SMFM stated (Louwen et al., 2025[73]): “At this time, the weight of scientific evidence that acetaminophen use during pregnancy causes an increased risk for autism or ADHD is simply inconclusive. It is important to understand that untreated fever and pain during pregnancy carry significant maternal and infant health risks. RCOG affirmed that paracetamol “remains the safest option for pregnant women” (BJOG, 2025[13]). EMA stated September 23, 2025 (EMA, 2025[36]): “There was no new evidence that would require changes to the region's current recommendations for the use of paracetamol during pregnancy... paracetamol could be used during pregnancy when needed, though at the lowest effective dose and frequency”.

These organizational responses shared common elements (BJOG, 2025[13]; Louwen et al., 2025[73]; EMA, 2025[36]; Szubert et al., 2025[98]): (1) Emphasis on scientific consensus; (2) Explicit reference to high-quality sibling-controlled studies; (3) Discussion of risks from untreated conditions; (4) Measured rather than alarmist language; and (5) Implicit criticism of governmental overreach.

#### Scientific expert commentary

International experts interviewed by Nature provided consistent assessments (Pearson and Ledford, 2025[84]). Monique Botha (Durham University): “There is no robust evidence or convincing studies to suggest there is any causal relationship, and any conclusions being drawn to the contrary are often motivated, under-evidenced and unsupported by the most robust methods” (Pearson and Ledford, 2025[84]). James Cusack (Autistica, London): “There is no definitive evidence to suggest that paracetamol use in mothers is a cause of autism, and when you see any associations, they are very, very small... At the heart of this is people trying to look for simple answers to complex problems” (Pearson and Ledford, 2025[84]). Sura Alwan (University of British Columbia): “The evidence does not support a causal link between acetaminophen or vaccines and autism... Suggesting otherwise may fuel misinformation and undermine confidence in safe treatments and immunizations” (Pearson and Ledford, 2025[84]). Helen Tager-Flusberg (Boston University): “The better-controlled studies are less likely to find even a small risk… And even then, what we're talking about is a minor association. We do not think that taking acetaminophen is in any way contributing to actually causing autism” (Pearson and Ledford, 2025[84]).

### Epidemiological evidence: What the science actually shows

#### High-quality sibling-control studies

*Swedish National Cohort:* This study represents the highest-quality evidence available (Ahlqvist et al., 2024[3]). Researchers examined 2,480,797 children born in Sweden between 1995 and 2019, using both prescription records and antenatal documentation (Ahlqvist et al., 2024[3]). Approximately 186,000 children were exposed to paracetamol during pregnancy (Ahlqvist et al., 2024[3]). Conventional analysis identified modest associations: 1.42 % of exposed children diagnosed with autism compared to 1.33 % of unexposed, a difference of 0.09 percentage points (Ahlqvist et al., 2024[3]). However, sibling-controlled analysis comparing siblings born to the same mother found no associations with autism (hazard ratio (HR) 0.99, 95 % confidence interval (CI) 0.93-1.05), ADHD (HR 1.03, 95 % CI 0.99-1.07), or intellectual disability (HR 0.99, 95 % CI 0.89-1.10) (Ahlqvist et al., 2024[3]). The authors concluded: “In sibling comparisons, associations with neurodevelopmental outcomes were attenuated to no associations, suggesting that associations in the general population may have been confounded” (Ahlqvist et al., 2024[3]). This pattern indicates that genetic and familial factors shared by siblings explain apparent associations (Ahlqvist et al., 2024[3]; D'Onofrio et al., 2013[31]).

*Japanese National Cohort:* This study examined 200,000 children using similar methodology (Okubo et al., 2026[82]). Initial analyses reported small associations. Sibling-controlled comparisons found no increased risks of autism or ADHD associated with maternal paracetamol use (Okubo et al., 2026[82]). The consistency of null findings across two independent populations strengthens causal inference (Ahlqvist et al., 2024[3]; Okubo et al., 2026[82]). Although conducted in Sweden and Japan, the consistency of null findings across two distinct healthcare systems and ethnic populations strengthens confidence in the generalizability of the results.

#### Importance of sibling-control designs in controlling unmeasured familial confounding

Sibling-control designs represent a powerful methodological approach for addressing unmeasured confounding (D'Onofrio et al., 2013[31]; Sjölander and Zetterqvist, 2017[96]; Tyrrell et al., 2012[102]). These studies compare siblings born to the same mother. This controls for all genetic and environmental factors shared within families (Sjölander and Zetterqvist, 2017[96]). This includes (D'Onofrio et al., 2013[31]; Tyrrell et al., 2012[102]): (1) Shared genetic variants influencing both maternal pain/illness and child neurodevelopment; (2) Stable environmental factors (socioeconomic status, parental education, neighborhood); (3) Parental behaviors and characteristics; (4) Health care access and utilization patterns. When conventional analyses show associations but sibling-controlled analyses show null findings, this strongly suggests confounding rather than causation (Ahlqvist et al., 2024[3]; Okubo et al., 2026[82]; Sjölander and Zetterqvist, 2017[96]). This methodological point is critical but was absent from the September 2025 governmental announcement (Horton, 2025[53]; Lavery, 2025[64]; Pearson and Ledford, 2025[84]).

#### Limitations of conventional observational studies

Earlier observational studies reporting associations suffered from critical methodological limitations (Bauer et al., 2018[9]; Liew et al., 2014[70]; VanderWeele and Ding, 2017[104]; Ystrom et al., 2017[110]): (1) Confounding by indication: Women use paracetamol because of fever, pain, or illness. These conditions may independently affect neurodevelopment (Hernán et al., 2004[49]; VanderWeele and Ding, 2017[104]); (2) Genetic confounding: Maternal genetic factors influence both medication use and offspring neurodevelopmental risk (D'Onofrio et al., 2013[31]; Tyrrell et al., 2012[102]); (3) Measurement error: Reliance on maternal recall introduces bias (VanderWeele and Ding, 2017[104]); and (4) Residual confounding: Even extensive covariate adjustment cannot address unmeasured factors (Hernán et al., 2004[49]; Sjölander and Zetterqvist, 2017[96]).

#### Recent systematic reviews: Conflicting interpretations

Two systematic reviews reached opposing conclusions, illustrating how evidence synthesis methodology matters (Prada et al., 2025[88]; Talge, 2020[100]). The Navigation Guide review applied environmental health methodology and concluded evidence supports associations (Prada et al., 2025[88]). However, this review (Prada et al., 2025[88]): (1) Counted a number of studies showing associations without adequately weighting methodological quality; (2) Treated the two sibling-controlled studies as merely “two among many” rather than recognizing their superior design; (3) Applied frameworks developed for environmental toxicants to pharmaceutical questions where confounding by indication is paramount. The Danish review applied pharmaco-epidemiological standards and concluded: “In utero exposure to acetaminophen is unlikely to confer a clinically important increased risk” of neurodevelopmental disorders (Prada et al., 2025[88]). This review (Prada et al., 2025[88]): (1) Weighted studies by methodological quality; (2) Emphasized sibling-controlled findings; (3) Acknowledged persistent confounding in observational studies. The September 2025 governmental announcement selectively cited the Navigation Guide review (Prada et al., 2025[88]) while minimizing higher-quality pharmaco-epidemiological evidence pointing to the opposite conclusion (Lavery, 2025[64]).

### Established risks of untreated maternal fever and pain

The governmental recommendation to avoid paracetamol ignores well-established risks of untreated conditions, such as maternal fever, and chronic pain (Bjelland et al., 2003[12]; Dreier et al., 2014[32]; Horton, 2025[53]; Polifka and Friedman, 2002[87]). Concerning maternal fever, a 2017 systematic review reported substantial evidence that maternal fever increases risks of neural tube defects, congenital heart defects, and oral clefts (Polifka and Friedman, 2002[87]). A 2014 meta-analysis confirmed these associations across multiple populations (Dreier et al., 2014[32]). Mechanisms involve direct teratogenic effects of hyperthermia on developing tissues (Edwards, 2006[34]; Polifka and Friedman, 2002[87]). The CDC classifies fever > 38 °C during pregnancy as an urgent warning sign (Wisner, 2021[107]). Regarding chronic pain which affects 20-30 % of pregnancies (Bjelland et al., 2003[12]; Gutke et al., 2007[46]), it appears that severe untreated pain contributes to depression, anxiety, sleep disturbance, and reduced functioning (Bjelland et al., 2003[12]; Gutke et al., 2007[46]). Maternal depression associates with preterm birth, low birthweight, and offspring behavioral problems (Grote et al., 2010[44]). Migraine during pregnancy associates with preeclampsia and gestational hypertension (Negro et al., 2017[80]). Finally, since there are no safer alternatives (i.e., Nonsteroidal anti-inflammatory drugs carry known risks of miscarriage and fetal complications (Antonucci et al., 2012[5]; Daniel et al., 2014[24]), and opioids present risks of neonatal abstinence syndrome (Kocherlakota, 2014[61])), paracetamol represents the only recommended antipyretic/analgesic (Louwen et al., 2025[73]; Luxey et al., 2025[74]; Szubert et al., 2025[98]).

### The MMR-autism controversy: Historical parallel

The September 2025 paracetamol announcement replicates the MMR-autism crisis (Deer, 2011[27]; Godlee et al., 2011[41]; Leask and Chapman, 1998[66]; Wakefield et al., 1998[105]).

#### The Wakefield fraud

In February 1998, Andrew Wakefield published a paper in The Lancet claiming MMR vaccine caused autism (Wakefield et al., 1998[105]). The study was fraudulent (Lindsay, 2021[72]; Wakefield et al., 1998[105]): Data were fabricated, cases cherry-picked, and Wakefield had undisclosed financial conflicts (Deer, 2020[28]). At a press conference, Wakefield made categorical claims despite the paper's limitations (Wakefield et al., 1998[105]). Despite immediate skepticism, media coverage amplified the controversy (Balog-Way and McComas, 2025[8]; Leask and Chapman, 1998[66]). Vaccination rates declined sharply in the UK (Jansen et al., 2003[56]; Salmon et al., 2006[93]). Measles outbreaks resulted (Jansen et al., 2003[56]). The paper was retracted in 2010 and Wakefield struck off the medical register (Deer, 2020[28]; Wakefield et al., 1998[105]). Multiple large studies consistently highlighted no MMR-autism association (Hviid et al., 2019[55]; Madsen et al., 2002[75]; Taylor et al., 2014[101]). A 2019 Danish study of 657,461 children reported no relationship even in high-risk children (Hviid et al., 2019[55]). The scientific consensus is unambiguous (Taylor et al., 2014[101]).

#### Communication failures

Analysis identified the following specific MMR communication failures (Balog-Way and McComas, 2025[8]; Leask et al., 2010[65]; Leask and Chapman, 1998[66]):

1. False balance: Media presented equal weight to Wakefield's claims despite overwhelming contradictory evidence (Balog-Way and McComas, 2025[8]; Dixon and Clarke, 2013[30]);
2. Categorical claims: Wakefield and media made causal statements despite weak evidence (Leask and Chapman, 1998[66]; Wakefield et al., 1998[105]);
3. Inadequate government response: Early responses emphasized authority rather than engaging concerns (Leask et al., 2010[65]; Leask and Chapman, 1998[66]);
4. Ignoring methodological quality: Media did not distinguish high-quality from low-quality studies (Balog-Way and McComas, 2025[8]; Dixon and Clarke, 2013[30]); and
5. Trust erosion: Defensive responses damaged credibility (Larson et al., 2011[63]; Leask et al., 2010[65]).

These failures caused lasting vaccine hesitancy and preventable deaths (Dube et al., 2015[33]; Jansen et al., 2003[56]; Salmon et al., 2006[93]).

### Comparative analysis: MMR and paracetamol controversies

Table 2(Tab. 2) (References in Table 2: Ahlqvist et al., 2024[3]; Antonucci et al., 2012[5]; Balog-Way and McComas, 2025[8]; Bauer et al., 2018[9]; Bjelland et al., 2003[12]; BJOG, 2025[13]; Daniel et al., 2014[24]; Deer, 2020[28]; Dixon and Clarke, 2013[30]; Dreier et al., 2014[32]; Dube et al., 2015[33]; EMA, 2025[36]; Gulati et al., 2025[45]; Hernán et al., 2004[49]; Horton, 2025[53]; Hviid et al., 2019[55]; Jansen et al., 2003[56]; Kocherlakota, 2014[61]; Larson et al., 2011[63]; Lavery, 2025[64]; Leask and Chapman, 1998[66]; Leask et al., 2010[65]; Liew et al., 2014[70]; Louwen et al., 2025[73]; Madsen et al., 2002[75]; Okubo et al., 2026[82]; Pearson and Ledford, 2025[84]; Polifka and Friedman, 2002[87]; Salmon et al., 2006[93]; Szubert et al., 2025[98]; Taylor et al., 2014[101]; Tyrrell et al., 2012[102]; VanderWeele and Ding, 2017[104]; Wakefield et al., 1998[105]; Ystrom et al., 2017[110]) systematically compares the two controversies, revealing striking parallels. The parallels are comprehensive and disturbing. The September 2025 governmental announcement replicates every major MMR communication failure despite almost 27 years to learn from that crisis (Godlee et al., 2011[41]; Horton, 2025[53]; Lavery, 2025[64]; Leask and Chapman, 1998[66]; Pearson and Ledford, 2025[84]; Wakefield et al., 1998[105]).

### Media and public response: Initial observations

Analysis identified Media coverage of the September 2025 announcement exhibited predictable patterns (Horton, 2025[53]; Ramos, 2025[90]; Pearson and Ledford, 2025[84]). Major outlets quoted both governmental warnings and scientific experts' rebuttals, often presenting equal weight to both perspectives (Ramos, 2025[90]). Headlines frequently emphasized controversy rather than scientific consensus (Ramos, 2025[90]). Social media rapidly amplified alarm (Merchant and Asch, 2018[77]). The Eurovision fact-checking network analyzed Governmental claims about Cuba's autism rates and found it unsupported (Ramos, 2025[90]): Cuba has paracetamol (though shortages exist), autism prevalence data are limited and non-comparable, and no evidence links paracetamol access to autism rates (Ramos, 2025[90]). Yet this fact-check received less attention than the original claim (Eysenbach, 2009[38]; Ramos, 2025[90]). Parent forums and social media groups showed confusion and concern (Merchant and Asch, 2018[77]). Many pregnant individuals expressed uncertainty about whether to take paracetamol for fever or pain (Merchant and Asch, 2018[77]). This creates immediate risk of harm from untreated conditions (Bjelland et al., 2003[12]; Polifka and Friedman, 2002[87]).

## Discussion

The September 22, 2025 governmental announcement regarding paracetamol and autism represents a predictable and preventable failure to apply lessons from the MMR-autism crisis (Godlee et al., 2011[41]; Horton, 2025[53]; Leask and Chapman, 1998[66]; Pearson and Ledford, 2025[84]; Wakefield et al., 1998[105]). This real-time event demonstrates how governmental overreach, selective citation of evidence, and inflammatory rhetoric can undermine public health despite scientific consensus to the contrary (Horton, 2025[53]; Louwen et al., 2025[73]; Pearson and Ledford, 2025[84]; Szubert et al., 2025[98]). We discussed the implications for evidence-based medicine, clinical practice, and public trust.

### The evidence does not support causal claims

The highest-quality epidemiological evidence provides strong evidence against causal relationships between prenatal paracetamol exposure and neurodevelopmental disorders (Ahlqvist et al., 2024[3]; Okubo et al., 2026[82]). The Swedish and Japanese sibling-controlled studies, examining nearly 2.7 million children combined, consistently found null associations once genetic and familial confounding were addressed (Ahlqvist et al., 2024[3]; Okubo et al., 2026[82]). As epidemiologist Viktor Ahlqvist noted, the sibling-control methodology “breaks the link” between unmeasured confounders and apparent associations (Ahlqvist et al., 2024[3]; Pearson and Ledford, 2025[84]). The divergence between conventional and sibling-controlled analyses has clear interpretation (Ahlqvist et al., 2024[3]; D'Onofrio et al., 2013[31]; Okubo et al., 2026[82]; Sjölander and Zetterqvist, 2017[96]; Tyrrell et al., 2012[102]). When conventional analyses show associations but sibling comparisons show null findings, confounding explains the conventional associations (Sjölander and Zetterqvist, 2017[96]). This is not speculative interpretation. It is fundamental epidemiological reasoning supported by genetic epidemiology (D'Onofrio et al., 2013[31]; Sjölander and Zetterqvist, 2017[96]; Tyrrell et al., 2012[102]). Maternal genetic variants influence pain sensitivity, inflammatory conditions, and medication-seeking behavior (Tyrrell et al., 2012[102]). These same variants transmit to offspring and may influence neurodevelopmental phenotypes through pleiotropy (D'Onofrio et al., 2013[31]; Tyrrell et al., 2012[102]).

Previous systematic reviews emphasizing the quantity of studies showing associations, like the Navigation Guide review (Prada et al., 2025[88]), fall into a well-recognized error in evidence synthesis: treating all studies as equally informative regardless of methodological quality (Guyatt et al., 2008[47]; Schünemann et al., 2019[95]; Prada et al., 2025[88]). The principle that “the plural of anecdote is not data” applies equally to observational studies with severe confounding (Guyatt et al., 2008[47]). Numerous flawed studies do not outweigh fewer methodologically superior studies (Guyatt et al., 2008[47]; Schünemann et al., 2019[95]). The FDA's September 22, 2025 statements acknowledged this reality, noting “a causal relationship has not been established, and there are contrary studies in the scientific literature” (FDA, 2025[39]). The White House announcement ignored this nuance (Horton, 2025[53]; Lavery, 2025[64]; Pearson and Ledford, 2025[84]).

### Ignoring risks of untreated conditions

The governmental recommendation to avoid paracetamol ignores established harms from untreated maternal fever and pain (Bjelland et al., 2003[12]; Dreier et al., 2014[32]; Horton, 2025[53]; Polifka and Friedman, 2002[87]). This represents dangerous asymmetry: emphasizing uncertain medication risks while dismissing certain risks of untreated conditions (Louwen et al., 2025[73]; Szubert et al., 2025[98]). Maternal fever, particularly in the first trimester, substantially increases risks of major congenital malformations (Dreier et al., 2014[32]; Polifka and Friedman, 2002[87]). A 2017 systematic review identified consistent evidence across multiple populations that fever increases risks of neural tube defects, heart defects, and facial clefts (Polifka and Friedman, 2002[87]). These are not minor risks. Neural tube defects cause lifelong disability (Atta et al., 2016[7]). The biological mechanism, direct teratogenic effects of hyperthermia, is well-established (Edwards, 2006[34]; Polifka and Friedman, 2002[87]).

Untreated pain contributes to maternal depression and anxiety (Bjelland et al., 2003[12]; Gutke et al., 2007[46]), both of which are independently associated with adverse pregnancy outcomes (Grote et al., 2010[44]). Chronic pain affects 20-30 % of pregnancies, creating substantial morbidity (Bjelland et al., 2003[12]; Gutke et al., 2007[46]). For migraine sufferers, most effective treatments are contraindicated in pregnancy, leaving paracetamol as one of few options despite its limited efficacy (Nappi et al., 2022[79]; Negro et al., 2017[80]). The President's advice to “fight like hell not to take” paracetamol creates a false dichotomy between medication use and enduring suffering (Horton, 2025[53]; Pearson and Ledford, 2025[84]). This ignores decades of clinical experience showing appropriate paracetamol use balances treatment of maternal conditions with fetal safety (Louwen et al., 2025;[73] Luxey et al., 2025[74]; Szubert et al., 2025[98]).

### Biological plausibility arguments: Potential neurotoxic and developmental mechanisms of prenatal paracetamol exposure

High-dose animal experiments and in-vitro studies have suggested potential effects of paracetamol on endocrine function, anandamide signaling, and oxidative stress (OS) (Sarzi-Puttini et al., 2024[94]). However, these models rely on exposures far exceeding human therapeutic levels and lack consistent replication at clinically relevant doses. Crucially, biological plausibility cannot outweigh robust epidemiological evidence showing no associations after adequate confounding control - a principle firmly established in teratology and pharmaco-epidemiology. While large sibling-controlled cohorts do not indicate a causal link between typical prenatal paracetamol use and autism or ADHD, experimental and translational findings suggest possible neurodevelopmental pathways that merit attention in cases of high or prolonged exposure. These mechanistic insights do not demonstrate causality in humans but offer biological context for interpreting associative epidemiological findings (Ahlqvist et al., 2024[3]) (Table 3(Tab. 3)).

#### Placental transfer, fetal exposure, and oxidative stress

Paracetamol readily crosses the placenta, achieving fetal plasma concentrations similar to maternal levels within a short time after dosing, which ensures direct exposure of the developing brain to parent drug and metabolites (Conings et al., 2019[22]; Klein et al., 2023[60]). At therapeutic doses most paracetamol undergoes glucuronidation and sulfation, but a fraction is bioactivated by cytochrome P450 to N‑acetyl‑p‑benzoquinone imine (NAPQI), which is detoxified by glutathione; under conditions of reduced antioxidant capacity or repeated dosing, NAPQI can accumulate and induce OS (Bührer et al., 2021[18]; Klein et al., 2023[60]; Mazaleuskaya et al., 2015[76]). OS during late gestation, when cortical surface area expands rapidly and synaptogenesis accelerates, may alter microglial-neuronal crosstalk, dendritic arborization, and myelination, with potential long‑term consequences for attention and behavior (Huang et al., 2024[54]; Klein et al., 2023[60]). Umbilical‑cord biomarker studies have reported that higher unmetabolized acetaminophen and OS markers (e.g., 8‑hydroxy‑deoxyguanosine) coexist in some exposed pregnancies, supporting a biologically coherent stress pathway in susceptible dyads (Anand et al., 2021[4]; Klein et al., 2023[60]).

#### Endocannabinoid and neurotransmitter modulation

Paracetamol's analgesic action partly involves conversion to AM404 (N-arachidonoyl-phenolamine), an active metabolite that inhibits anandamide reuptake and modulates cannabinoid receptor type 1 (CB1) signaling in the brain (Hogestatt et al., 2005[52]; Philippot et al., 2018[86]). The endocannabinoid system regulates progenitor proliferation, neuronal migration, axonal pathfinding, and synaptic pruning in early development; perturbation during critical windows in rodents produces long‑lasting effects on locomotion, anxiety, and cognitive performance (Harkany et al., 2007[48]; Philippot et al., 2018[86]). Experimental mouse models show that neonatal or gestational paracetamol exposure, especially in combination with a CB1 agonist, can disrupt expression of CB1‑related genes and neurotrophin pathways and is associated with altered exploratory behavior and social interaction in adulthood (Philippot et al., 2018[86]). Additional studies report sex‑specific changes in prefrontal cortex gene expression related to glutathione metabolism, cytochrome P450 enzymes, deoxyribonucleic acid (DNA) damage response, and immune signaling after developmental paracetamol exposure, paralleling subtle alterations in anxiety‑like behavior and activity patterns (Caballero et al., 2007[19]; Liew and Ernst, 2021[69]; Philippot et al., 2018[86]). These findings suggest that interactions between endocannabinoid modulation, OS, and sex hormones may contribute to heterogeneous neurobehavioral outcomes in animal models, although translational relevance to standard human dosing remains uncertain (Caballero et al., 2007[19]; Liew and Ernst, 2021[69]; Philippot et al., 2018[86]).

#### Epigenetic mechanisms and developmental programming

Fetal neurodevelopment is highly sensitive to epigenetic programming, and xenobiotic exposures during gestation can induce persistent changes in DNA methylation, histone modifications, and non‑coding ribonucleic acid (RNA) expression that influence brain structure and function across the life course (Herrington et al., 2022[50]; Kundakovic and Champagne, 2015[62]). In cord‑blood epigenome‑wide association studies, long‑term prenatal paracetamol use (e.g., ≥ 20 days) has been associated with differential DNA methylation at thousands of CpG (i.e., cytosine-phosphate-guanine) sites in genes implicated in OS regulation, synaptic transmission, and neurodevelopment among children who later developed ADHD (Gervin et al., 2017[40]; Herrington et al., 2022[50]). A complementary study in extremely preterm infants identified that reported maternal acetaminophen use was associated with altered placental methylation signatures, with evidence of sex‑specific patterns and enrichment in pathways related to immune signaling and neuronal differentiation (Addo et al., 2019[2]; Herrington et al., 2022[50]). Animal experiments further indicate that repeated developmental paracetamol exposure can modify global and gene‑specific methylation patterns in the brain, paralleling changes in learning and memory performance, which supports a dose-response relationship between exposure, epigenetic disruption, and behavior in susceptible models (Raciti and Ceccatelli, 2018[89]). These data are consistent with a scenario in which paracetamol does not uniformly cause neurodevelopmental disorders but may, under particular combinations of dose, timing, and genetic background, contribute to subtle shifts in neurodevelopmental trajectories via epigenetic mechanisms (Bauer et al., 2021[10]; Rice and Barone, 2000[91]).

#### Integration with human epidemiology

Mechanistic observations need to be interpreted alongside human cohort data that show attenuation of autism and ADHD associations when family‑level confounding is rigorously controlled (Liew et al., 2016[71]). Sibling‑comparison studies suggest that shared genetic and environmental factors explain much of the crude association between prenatal paracetamol use and neurodevelopmental diagnoses, yet they cannot completely exclude risk in subgroups with prolonged high‑dose exposure or specific susceptibility profiles (Brandlistuen et al., 2013[16]). A coherent interpretation is that, for typical short‑course use at recommended doses, any direct neurotoxic effect is likely very small relative to background familial risk and the harms of untreated fever or pain, whereas prolonged or repeated high‑dose courses could, in theory, interact with OS burden and epigenetic vulnerability in a minority of pregnancies (Bauer et al., 2021[10]). From a clinical perspective, this mechanistic evidence reinforces existing guidance to use paracetamol at the lowest effective dose for the shortest possible duration, to avoid repeated high‑dose self‑medication, and to carefully document indication, timing, and cumulative exposure during pregnancy (Dathe and Schaefer, 2019[26]).

### Governmental overreach and the politicization of evidence

The September 2025 events represent unprecedented governmental interference with evidence-based medicine (Horton, 2025[53]; Lavery, 2025[64]; Pearson and Ledford, 2025[84]). While previous administrations have occasionally misrepresented scientific evidence, direct categorical warnings contradicting professional medical consensus and the highest-quality studies are extraordinary (Horton, 2025[53]; Pearson and Ledford, 2025[84]).

The involvement of HHS Secretary Robert F. Kennedy Jr., known for promoting anti-vaccine theories, raises concerns about ideologically motivated policy (Gulati et al., 2025[45]; Horton, 2025[53]; Pearson and Ledford, 2025[84]). Kennedy's March 2025 announcement of a CDC study examining vaccine-autism links, despite decades of definitive research showing no association, suggests a pattern of using governmental authority to challenge scientific consensus (Gulati et al., 2025[45]; Hviid et al., 2019[55]; Madsen et al., 2002[75]; Taylor et al., 2014[101]).

The divergence between the White House's categorical warnings and the FDA's nuanced advisory reveals institutional tensions (Horton, 2025[53]; Pearson and Ledford, 2025[84]; FDA, 2025[39]). The FDA, bound by scientific standards and legal requirements, acknowledged uncertainty (FDA, 2025[39]). The White House, facing no such constraints, made categorical claims (Horton, 2025[53]; Pearson and Ledford, 2025[84]). This undermines the FDA's credibility and the principle of science-based regulation (Jasanoff, 2013[57]). Comparisons to the MMR crisis reveal an important distinction (Godlee et al., 2011[41]; Leask and Chapman, 1998[66]; Wakefield et al., 1998[105]). Wakefield was a fringe researcher whose claims contradicted governmental positions (Wakefield et al., 1998[105]). In 2025, the governmental position contradicts scientific consensus (Horton, 2025[53]; Louwen et al., 2025[73]; Pearson and Ledford, 2025[84]; Szubert et al., 2025[98]). This inversion is more dangerous because it exploits governmental authority to legitimize scientifically unsupported claims (Horton, 2025[53]; Pearson and Ledford, 2025[84]).

It is noteworthy that several authors of the 2021 precautionary consensus statement (Bauer et al., 2021[10]) and some researchers reporting positive associations have served as paid experts in ongoing U.S. product-liability litigation against acetaminophen manufacturers. While this does not invalidate individual studies, it underscores the importance of prioritizing studies without such conflicts and those using the strongest confounding-control designs.

### Media failures: False balance revisited

Media coverage of the September 2025 announcement exhibits the false balance that characterized MMR reporting (Balog-Way and McComas, 2025[8]; Dixon and Clarke, 2013[30]; Leask and Chapman, 1998[66]; Ramos, 2025[90]). Presenting the President's warnings and scientists' rebuttals as equally valid “sides” misleads the public about the state of evidence (Ramos, 2025[90]; Pearson and Ledford, 2025[84]). The false balance problem is not new (Balog-Way and McComas, 2025[8]; Dixon and Clarke, 2013[30]). Boyce's 2007 analysis of MMR coverage documented how “balanced” journalism created misleading impressions of scientific controversy where none existed (Balog-Way and McComas, 2025[8]). The same pattern emerged in climate change coverage, where equal time to climate sceptics misrepresented overwhelming scientific consensus (Boykoff and Boykoff, 2004[14]).

Effective science journalism requires distinguishing genuine scientific debate from disagreement between evidence and political claims (Balog-Way and McComas, 2025[8]; Boykoff and Boykoff, 2004[14]; Dixon and Clarke, 2013[30]). When the best evidence points one direction but political figures claim another, journalists must communicate this clearly rather than presenting false equivalence (Boykoff and Boykoff, 2004[14]). The Nature article by Pearson and Ledford provides a model: it prominently quoted multiple independent experts emphasizing lack of causal evidence while contextualizing the governmental announcement (Pearson and Ledford, 2025[84]). However, not all outlets followed this standard (Ramos, 2025[90]).

### The precautionary communication principle: A framework for clinicians

**Figure 2:**
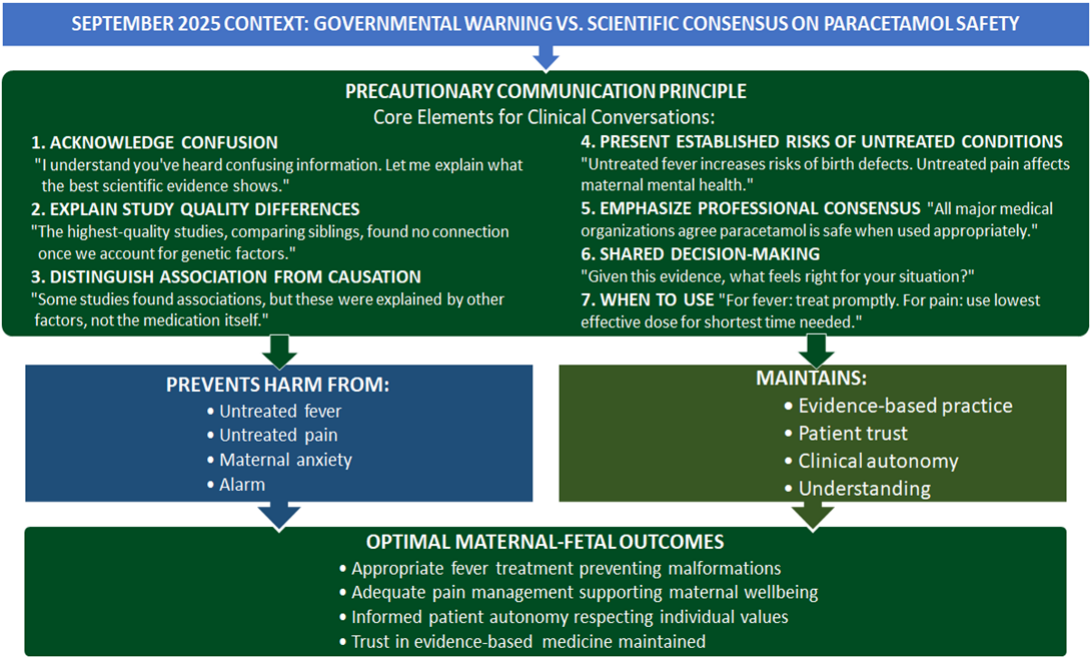
Precautionary Communication Principle for navigating paracetamol-autism controversy. Evidence-based framework integrating epidemiological hierarchy (sibling-controlled studies: Ahlqvist et al. 2024, n = 2.48 million; Okubo et al. 2026, n = 200,000), professional consensus (ACOG, SMFM, RCOG, EMA 2025 statements), and shared decision-making principles. Context: September 2025 governmental warnings contradicted scientific consensus; framework addresses political-scientific discordance while preserving clinical autonomy and patient agency. ACOG: American College of Obstetricians and Gynecologists; EMA: European Medicines Agency; RCOG: Royal College of Obstetricians and Gynaecologists; SMFM: Society for Maternal-Fetal Medicine

Clinicians now face confused and concerned patients asking about paracetamol safety (Louwen et al., 2025[73]; Szubert et al., 2025[98]). The Precautionary Communication Principle provides an evidence-based framework for these conversations (Figure 2(Fig. 2)). This framework emphasizes transparent communication about evidence quality while avoiding both false reassurance and disproportionate alarm (Louwen et al., 2025[73]; Szubert et al., 2025[98]). Key principles include (Elwyn et al., 2012[35]; Legare et al., 2008[67]):

1. *Acknowledge the confusion:* Patients have valid reasons for concern given governmental warnings (Horton, 2025[53]; Pearson and Ledford, 2025[84]). Dismissing these concerns alienates patients (Covello et al., 2001[23]; Leask et al., 2010[65]). Begin by validating uncertainty while promising to explain the evidence (Elwyn et al., 2012[35]).
2. *Explain study quality differences:* Most patients do not understand sibling-control designs (Ahlqvist et al., 2024[3]; Okubo et al., 2026[82]; Sjölander and Zetterqvist, 2017[96]). Use accessible language: “The best studies compared siblings where one was exposed to paracetamol and the other wasn't. Since siblings share genes and family environment, this controls for factors that confused earlier studies. These studies found no connection” (Ahlqvist et al., 2024[3]; Okubo et al., 2026[82]).
3. *Distinguish association from causation:* Many patients misunderstand that correlation does not imply causation (Guyatt et al., 2008[47]; Schünemann et al., 2019[95]). Use concrete examples: “Women who use paracetamol during pregnancy often have fevers or pain. These conditions might affect development regardless of medication. The sibling studies showed the medication itself isn't the problem” (Ahlqvist et al., 2024[3]; Okubo et al., 2026[82]; VanderWeele and Ding, 2017[104]).
4. *Present risks of untreated conditions:* Do not just emphasize medication safety. Explain established harms from untreated fever and pain (Bjelland et al., 2003[12]; Dreier et al., 2014[32]; Polifka and Friedman, 2002[87]). Use specific examples: “Untreated fever in early pregnancy increases risk of neural tube defects. These cause spina bifida. This risk is well-established, unlike uncertain medication risks” (Polifka and Friedman, 2002[87]).
5. *Cite professional consensus:* Emphasize that ACOG, SMFM, RCOG, and EMA all affirm paracetamol safety (BJOG, 2025[13]; Louwen et al., 2025[73]; EMA, 2025[36]; Szubert et al., 2025[98]). This provides institutional credibility: “Every major medical organization worldwide agrees paracetamol is safe for pregnancy when used appropriately” (Louwen et al., 2025[73]; EMA, 2025[36]; Szubert et al., 2025[98]).
6. *Employ shared decision-making:* Present evidence but respect autonomy (Charles et al., 1997[20]; Elwyn et al., 2012[35]). “Given this evidence, how do you feel about using paracetamol if needed?” allows patients to integrate information with their values (Charles et al., 1997[20]).
7. *Provide clear guidance on use:* “For fever, treat promptly with paracetamol. For pain, use the lowest dose that helps, for the shortest time needed” (Louwen et al., 2025[73]; Luxey et al., 2025[74]; Szubert et al., 2025[98]). This follows professional guidelines (Louwen et al., 2025[73]; Szubert et al., 2025[98]).

### Broader implications for evidence-based medicine

The September 2025 events threaten evidence-based medicine's foundation (Horton, 2025[53]; Louwen et al., 2025[73]; Pearson and Ledford, 2025[84]; Szubert et al., 2025[98]). If governmental authorities can override scientific consensus with categorical warnings based on selective evidence, the entire enterprise of evidence-based practice is jeopardized (Jasanoff, 2013[57]; Sackett, 1998[92]). Evidence-based medicine rests on hierarchies of evidence quality and systematic assessment of bias (Guyatt et al., 2008[47]; Sackett, 1998[92]; Schünemann et al., 2019[95]). Sibling-controlled studies represent methodological innovation specifically designed to address confounding that plagues conventional observational studies (Sjölander and Zetterqvist, 2017[96]). Dismissing such studies in favor of lower-quality evidence with greater potential for bias represents a fundamental rejection of evidence-based principles (Guyatt et al., 2008[47]; Schünemann et al., 2019[95]).

The September 2025 announcement also threatens trust in regulatory institutions (Jasanoff, 2013[57]). The FDA's credibility depends on scientific independence from political pressure (Jasanoff, 2013[57]). When FDA statements diverge from White House messaging, this creates institutional confusion (Horton, 2025[53]; Pearson and Ledford, 2025[84]; FDA, 2025[39]). Future administrations inheriting this precedent may similarly override scientific consensus (Jasanoff, 2013[57]).

The MMR crisis taught that restoring trust after it erodes is extraordinarily difficult (Dube et al., 2015[33]; Jansen et al., 2003[56]; Larson et al., 2011[63]; Salmon et al., 2006[93]). Vaccination rates declined sharply after Wakefield's paper and took years to recover (Jansen et al., 2003[56]). Some communities never fully recovered (Dube et al., 2015[33]; Salmon et al., 2006[93]). The September 2025 events risk similar lasting damage to trust in prenatal medication guidance (Louwen et al., 2025[73]; Szubert et al., 2025[98]).

### What should have been done: Learning from MMR

The MMR experience offers clear lessons that were ignored in September 2025 (Balog-Way and McComas, 2025[8]; Leask et al., 2010[65]; Leask and Chapman, 1998[66]). Effective crisis communication requires (Covello et al., 2001[23]; Leask et al., 2010[65]; Leask and Chapman, 1998[66]):

- *Early engagement:* Professional organizations should have engaged proactively before governmental announcement (Leask et al., 2010[65]; Leask and Chapman, 1998[66]). ACOG and SMFM issued rapid responses (Louwen et al., 2025[73]; Szubert et al., 2025[98]). Pre-emptive communication might have prevented crisis (Leask and Chapman, 1998[66]).
- *Emphasize methodological quality:* Public communication should explain why sibling-controlled studies provide better evidence than conventional studies (Ahlqvist et al., 2024[3]; Okubo et al., 2026[82]; Sjölander and Zetterqvist, 2017[96]). This requires accessible language (Covello et al., 2001[23]; Elwyn et al., 2012[35]).
- *Address emotions alongside facts:* Parents concerned about autism deserve empathy, not dismissal (Covello et al., 2001[23]; Leask et al., 2010[65]). Effective messaging validates concerns while explaining evidence (Covello et al., 2001[23]).
- *Avoid false balance:* Media must distinguish political claims from scientific consensus (Balog-Way and McComas, 2025[8]; Boykoff and Boykoff, 2004[14]; Dixon and Clarke, 2013[30]). Journalists should consult independent methodologists, not just political figures and advocacy groups (Boykoff and Boykoff, 2004[14]).
- *Maintain institutional credibility:* Regulatory agencies must resist political pressure (Jasanoff, 2013[57]). The FDA's measured advisory contrasting with White House warnings exemplifies this tension (Horton, 2025[53]; Pearson and Ledford, 2025[84]; FDA, 2025[39]).

### Limitations of this analysis

This review acknowledged several limitations. First, as a narrative review analyzing real-time events, we could not employ systematic review protocols with prospective registration (Green et al., 2006[42]). The September 2025 events remain ongoing. Additional information may emerge (Brownson et al., 2009[17]). Long-term impacts on patient behavior, clinical practice, and public trust cannot yet be quantified (Eysenbach, 2009[38]). Media analysis remains preliminary (Eysenbach, 2009[38]; Ramos, 2025[90]). Second, English language restriction may exclude relevant international perspectives (Morrison et al., 2012[78]). Third, authors' interpretation of events necessarily involved subjective judgment (Greenhalgh et al., 2018[43]). However, the core findings are not subjective. Governmental warnings contradict the highest-quality evidence and replicate MMR communication failures (Ahlqvist et al., 2024[3]; Horton, 2025[53]; Louwen et al., 2025[73]; Okubo et al., 2026[82]; Pearson and Ledford, 2025[84]; Szubert et al., 2025[98]). These are documentable facts (Ahlqvist et al., 2024[3]; Horton, 2025[53]; Okubo et al., 2026[82]; Pearson and Ledford, 2025[84]). Fourth, our review was concentrated almost exclusively on autism/ADHD and public communication, with minimal discussion of other fetal outcomes despite their relevance to overall risk-benefit assessment (e.g., hepatotoxicity (Wu et al., 2023[108]; Yoon et al., 2016[109]), renal effects (Dathe et al., 2019[25]; Leverrier-Penna et al., 2021[68]), endocrine disruption (Jegou, 2015[58]; Tadokoro-Cuccaro et al., 2022[99])). Fifth, the mechanistic and experimental data on potential neurotoxicity (e.g., OS, endocannabinoid disruption, endocrine and epigenetic pathways) are briefly mentioned, although these are heavily emphasized in precautionary arguments and recent reviews (Bührer et al., 2021[18]; Caballero et al., 2007[19]; Gervin et al., 2017[40]; Liew and Ernst, 2021[69]; Wu et al., 2023[108]). Moreover, animal and translational studies that suggest plausible neurodevelopmental effects at doses close to the therapeutic range were briefly mentioned in relation to “biological plausibility,” whereas recent experimental literature points to specific mechanistic hypotheses (e.g., CB1 modulation, OS, sex‑specific prefrontal transcriptomic changes, epigenetic programming) deserve more neutral discussion (Philippot et al., 2018[86]). Sixth, the focus on US political dynamics may limit international relevance.

## Conclusion

The September 2025 governmental warning against paracetamol use in pregnancy repeats the communication failures of the MMR-autism crisis. Despite decades of evidence on effective risk communication, authorities issued categorical warnings that contradict scientific consensus, relied on low-quality studies while dismissing high-quality evidence, used alarmist rhetoric, and failed to consider harms of inaction. The strongest evidence, two sibling-controlled studies covering nearly 2.7 million children, shows no causal link between prenatal paracetamol exposure and neurodevelopmental disorders once confounding is addressed. All major medical organizations affirm paracetamol's safety when used appropriately, and even the FDA acknowledged that causality has not been established. At the same time, the risks of untreated maternal fever and pain are well documented, including increased risks of congenital anomalies and maternal mental health disorders, and no safer alternatives exist. Advising avoidance forces pregnant individuals into harmful choices. Clinicians must respond through evidence-based shared decision-making, clearly distinguishing association from causation, presenting balanced risks of action and inaction, and referencing high-quality evidence and professional consensus. The episode highlights broader implications: political interference can undermine evidence-based medicine; lessons from past crises are not automatically applied; policymakers remain vulnerable to communication failures; and professional organizations play a critical role in defending scientific standards. Research should assess impacts on patient behavior, pregnancy outcomes, and communication strategies, while further studies could strengthen causal inference. Most importantly, the episode emphasizes the need for regulatory independence, institutional safeguards, and public understanding of science. As with the MMR crisis, failure to protect evidence-based practice risks preventable harm, this time potentially unfolding more rapidly.

## Notes

Ismail Dergaa and Nasr Chalghaf (High Institute of Sport and Physical Education of Gafsa, University of Gafsa 2100, Tunisia; Tel: +21658928407, E-mail: N.chalghaf@gmail.com) contributed equally as corresponding author.

## Declaration

### Artificial Intelligence (AI) - assisted technology

In preparing this manuscript, the authors used ChatGPT model 5 in September 2025 to refine passages, verify grammar, and enhance academic English. After using this tool, the authors thoroughly reviewed and edited the content and take full responsibility for the publication (Dergaa and Ben Saad, 2023[29]).

### Acknowledgments

The authors would like to express their sincere gratitude to the reviewer for his/her excellent feedback, which has substantially improved the quality of our work. His/her insightful comments and constructive suggestions were invaluable in refining our manuscript (Hidouri et al., 2024[51]).

### Ethics approval

Not applicable for this narrative review analyzing published literature and public statements.

### Consent for publication

All authors approved the final version for publication.

### Availability of data

All data analyzed are included in this article and publicly available sources cited in the reference list.

### Conflict of interest

The authors declare no competing interests.

### Funding

This narrative review received no specific funding.

### Authors' contributions

**Conceptualization:** H.B.S., A.E.O., I.D., C.B., N.C.; **Literature Search:** H.B.S., W.D., I.D.; **Methodology:** A.E.O., I.D., W.D., N.C.; **Validation:** H.B., H.I.C.; **Writing - Original Draft:** I.D.; **Writing - Review & Editing:** W.D., H.B.S., A.E.O., C.B., H.B.

## Figures and Tables

**Table 1:**
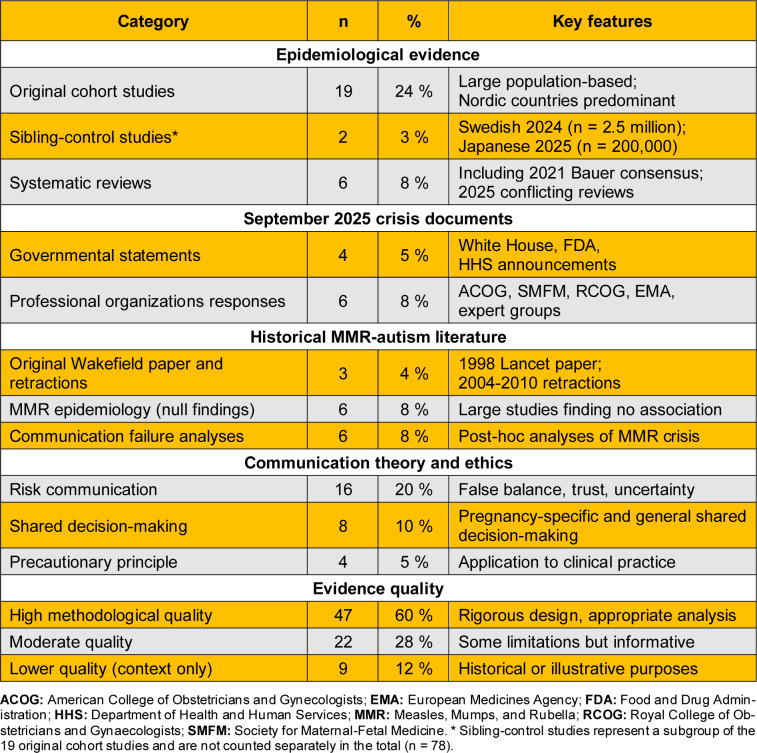
Characteristics of included literature (n = 78)

**Table 2:**
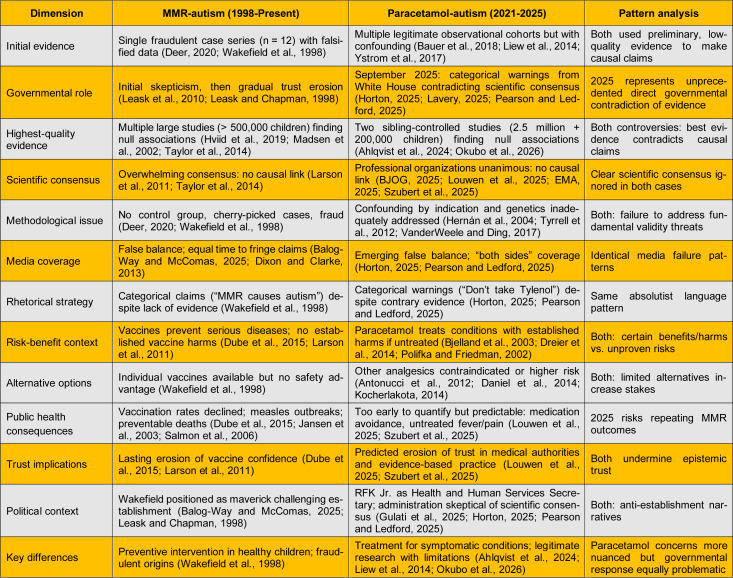
Comparative analysis of MMR (for measles, mumps, and rubella)-autism and paracetamol-autism controversies

**Table 3:**
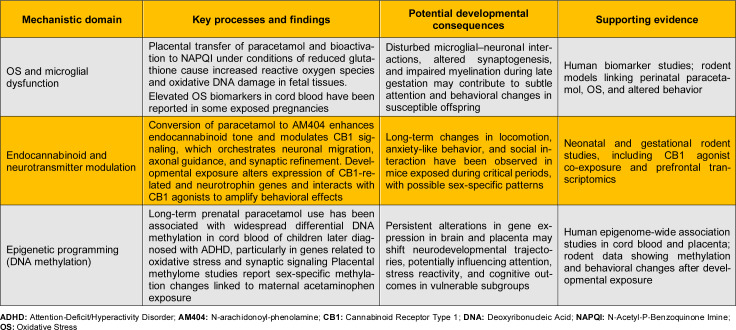
Potential neurotoxic and developmental mechanisms of prenatal paracetamol exposure

**Figure 1:**
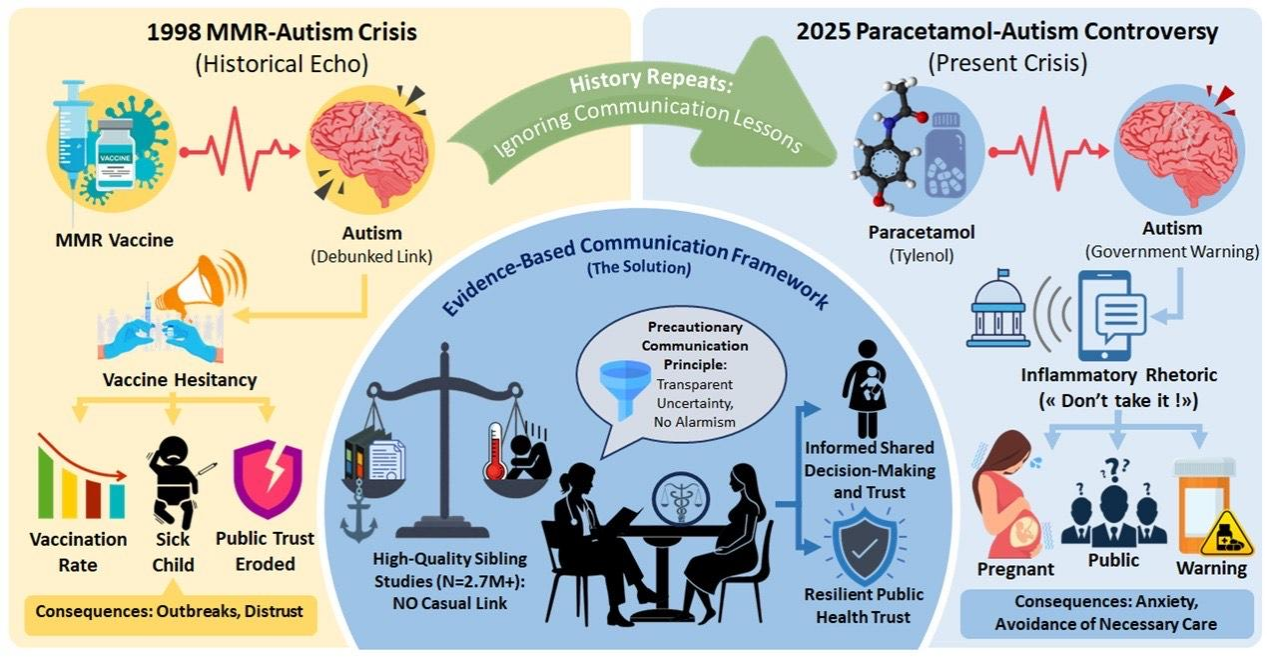
Graphical abstract

## References

[1] ACOG, American College of Obstetricians and Gynecologists (2025). Acetaminophen use in pregnancy and neurodevelopmental outcomes. https://www.acog.org/clinical/clinical-guidance/practice-advisory/articles/2025/09/acetaminophen-use-in-pregnancy-and-neurodevelopmental-outcomes.

[2] Addo KA, Bulka C, Dhingra R, Santos HP, Smeester L, O'Shea TM (2019). Acetaminophen use during pregnancy and DNA methylation in the placenta of the extremely low gestational age newborn (ELGAN) cohort. Environ Epigenet.

[3] Ahlqvist VH, Sjoqvist H, Dalman C, Karlsson H, Stephansson O, Johansson S (2024). Acetaminophen use during pregnancy and children's risk of autism, ADHD, and intellectual disability. JAMA.

[4] Anand NS, Raghavan R, Wang G, Hong X, Azuine RE, Pearson C (2021). Perinatal Acetaminophen exposure and childhood attention-deficit/hyperactivity disorder (ADHD): Exploring the role of umbilical cord plasma metabolites in oxidative stress pathways. Brain Sci.

[5] Antonucci R, Zaffanello M, Puxeddu E, Porcella A, Cuzzolin L, Pilloni MD (2012). Use of non-steroidal anti-inflammatory drugs in pregnancy: impact on the fetus and newborn. Curr Drug Metab.

[6] Atkins D, Best D, Briss PA, Eccles M, Falck-Ytter Y, Flottorp S (2004). Grading quality of evidence and strength of recommendations. BMJ.

[7] Atta CA, Fiest KM, Frolkis AD, Jette N, Pringsheim T, St Germaine-Smith C (2016). Global birth prevalence of spina bifida by folic acid fortification status: A systematic review and meta-analysis. Am J Public Health.

[8] Balog-Way D, McComas K (2025). Unpacking the risk of misinformation: a communication-based critique. Risk Anal.

[9] Bauer AZ, Kriebel D, Herbert MR, Bornehag CG, Swan SH (2018). Prenatal paracetamol exposure and child neurodevelopment: A review. Horm Behav.

[10] Bauer AZ, Swan SH, Kriebel D, Liew Z, Taylor HS, Bornehag CG (2021). Paracetamol use during pregnancy - a call for precautionary action. Nat Rev Endocrinol.

[11] Beauchamp T, Childress J (2019). Principles of biomedical ethics: Marking its fortieth anniversary. Am J Bioeth.

[12] Bjelland I, Tell GS, Vollset SE, Refsum H, Ueland PM (2003). Folate, vitamin B12, homocysteine, and the MTHFR 677C->T polymorphism in anxiety and depression: the Hordaland Homocysteine Study. Arch Gen Psychiatry.

[13] BJOG (2025). Supplement: Top Scoring Abstracts of the RCOG World Congress 2025, 23-25 June 2025 | ExCeL London. BJOG.

[14] Boykoff MT, Boykoff JM (2004). Balance as bias: global warming and the US prestige press. Glob Environ Change.

[15] Bramer WM, Rethlefsen ML, Kleijnen J, Franco OH (2017). Optimal database combinations for literature searches in systematic reviews: a prospective exploratory study. Syst Rev.

[16] Brandlistuen RE, Ystrom E, Nulman I, Koren G, Nordeng H (2013). Prenatal paracetamol exposure and child neurodevelopment: a sibling-controlled cohort study. Int J Epidemiol.

[17] Brownson RC, Chriqui JF, Stamatakis KA (2009). Understanding evidence-based public health policy. Am J Public Health.

[18] Bührer C, Endesfelder S, Scheuer T, Schmitz T (2021). Paracetamol (Acetaminophen) and the developing brain. Int J Mol Sci.

[19] Caballero FJ, Navarrete CM, Hess S, Fiebich BL, Appendino G, Macho A (2007). The acetaminophen-derived bioactive N-acylphenolamine AM404 inhibits NFAT by targeting nuclear regulatory events. Biochem Pharmacol.

[20] Charles C, Gafni A, Whelan T (1997). Shared decision-making in the medical encounter: what does it mean? (or it takes at least two to tango). Soc Sci Med.

[21] Chen MH, Pan TL, Wang PW, Hsu JW, Huang KL, Su TP (2019). Prenatal exposure to acetaminophen and the risk of attention-deficit/hyperactivity disorder: A nationwide study in Taiwan. J Clin Psychiatry.

[22] Conings S, Tseke F, Van den Broeck A, Qi B, Paulus J, Amant F (2019). Transplacental transport of paracetamol and its phase II metabolites using the ex vivo placenta perfusion model. Toxicol Appl Pharmacol.

[23] Covello VT, Peters RG, Wojtecki JG, Hyde RC (2001). Risk communication, the West Nile virus epidemic, and bioterrorism: responding to the communication challenges posed by the intentional or unintentional release of a pathogen in an urban setting. J Urban Health.

[24] Daniel S, Koren G, Lunenfeld E, Bilenko N, Ratzon R, Levy A (2014). Fetal exposure to nonsteroidal anti-inflammatory drugs and spontaneous abortions. CMAJ.

[25] Dathe K, Frank J, Padberg S, Hultzsch S, Meixner K, Beck E (2019). Negligible risk of prenatal ductus arteriosus closure or fetal renal impairment after third-trimester paracetamol use: evaluation of the German Embryotox cohort. BJOG.

[26] Dathe K, Schaefer C (2019). The use of medication in pregnancy. Dtsch Arztebl Int.

[27] Deer B (2011). How the case against the MMR vaccine was fixed. BMJ.

[28] Deer B (2020). The doctor who fooled the world.

[29] Dergaa I, Ben Saad H (2023). Artificial intelligence and promoting open access in academic publishing. Tunis Med.

[30] Dixon G, Clarke C (2013). The effect of falsely balanced reporting of the autism-vaccine controversy on vaccine safety perceptions and behavioral intentions. Health Educ Res.

[31] D'Onofrio BM, Lahey BB, Turkheimer E, Lichtenstein P (2013). Critical need for family-based, quasi-experimental designs in integrating genetic and social science research. Am J Public Health.

[32] Dreier JW, Andersen AM, Berg-Beckhoff G (2014). Systematic review and meta-analyses: fever in pregnancy and health impacts in the offspring. Pediatrics.

[33] Dube E, Vivion M, MacDonald NE (2015). Vaccine hesitancy, vaccine refusal and the anti-vaccine movement: influence, impact and implications. Expert Rev Vaccines.

[34] Edwards MJ (2006). Review: Hyperthermia and fever during pregnancy. Birth Defects Res A Clin Mol Teratol.

[35] Elwyn G, Frosch D, Thomson R, Joseph-Williams N, Lloyd A, Kinnersley P (2012). Shared decision making: a model for clinical practice. J Gen Intern Med.

[36] EMA, European Medicines Agency (2025). Use of paracetamol during pregnancy unchanged in the EU. https://www.ema.europa.eu/en/news/use-paracetamol-during-pregnancy-unchanged-eu.

[37] ENTIS, The European Network of Teratology Information Services (2021). Position statement on acetaminophen (paracetamol) in pregnancy. :.

[38] Eysenbach G (2009). Infodemiology and infoveillance: framework for an emerging set of public health informatics methods to analyze search, communication and publication behavior on the Internet. J Med Internet Res.

[39] FDA, U.S. Food and Drug Administration (2025). FDA responds to evidence of possible association between autism and acetaminophen use during pregnancy. https://www.fda.gov/news-events/press-announcements/fda-responds-evidence-possible-association-between-autism-and-acetaminophen-use-during-pregnancy.

[40] Gervin K, Nordeng H, Ystrom E, Reichborn-Kjennerud T, Lyle R (2017). Long-term prenatal exposure to paracetamol is associated with DNA methylation differences in children diagnosed with ADHD. Clin Epigenetics.

[41] Godlee F, Smith J, Marcovitch H (2011). Wakefield's article linking MMR vaccine and autism was fraudulent. BMJ.

[42] Green BN, Johnson CD, Adams A (2006). Writing narrative literature reviews for peer-reviewed journals: secrets of the trade. J Chiropr Med.

[43] Greenhalgh T, Thorne S, Malterud K (2018). Time to challenge the spurious hierarchy of systematic over narrative reviews?. Eur J Clin Invest.

[44] Grote NK, Bridge JA, Gavin AR, Melville JL, Iyengar S, Katon WJ (2010). A meta-analysis of depression during pregnancy and the risk of preterm birth, low birth weight, and intrauterine growth restriction. Arch Gen Psychiatry.

[45] Gulati S, Sharawat IK, Panda PK, Kothare SV (2025). The vaccine–autism connection: No link, still debate, and we are failing to learn the lessons. Autism.

[46] Gutke A, Josefsson A, Oberg B (2007). Pelvic girdle pain and lumbar pain in relation to postpartum depressive symptoms. Spine (Phila Pa 1976).

[47] Guyatt GH, Oxman AD, Vist GE, Kunz R, Falck-Ytter Y, Alonso-Coello P (2008). GRADE: an emerging consensus on rating quality of evidence and strength of recommendations. BMJ.

[48] Harkany T, Guzman M, Galve-Roperh I, Berghuis P, Devi LA, Mackie K (2007). The emerging functions of endocannabinoid signaling during CNS development. Trends Pharmacol Sci.

[49] Hernán MA, Hernández-Díaz S, Robins JM (2004). A structural approach to selection bias. Epidemiology.

[50] Herrington JA, Guss Darwich J, Harshaw C, Brigande AM, Leif EB, Currie PJ (2022). Elevated ghrelin alters the behavioral effects of perinatal acetaminophen exposure in rats. Dev Psychobiol.

[51] Hidouri S, Kamoun H, Salah S, Jellad A, Ben Saad H (2024). Key guidelines for responding to reviewers. F1000Res.

[52] Hogestatt ED, Jonsson BA, Ermund A, Andersson DA, Bjork H, Alexander JP (2005). Conversion of acetaminophen to the bioactive N-acylphenolamine AM404 via fatty acid amide hydrolase-dependent arachidonic acid conjugation in the nervous system. J Biol Chem.

[53] Horton R (2025). Offline: Those one should not forgive. The Lancet.

[54] Huang Y, Qiu F, Dziegielewska KM, Koehn LM, Habgood MD, Saunders NR (2024). Effects of paracetamol/acetaminophen on the expression of solute carriers (SLCs) in late-gestation fetal rat brain, choroid plexus and the placenta. Exp Physiol.

[55] Hviid A, Hansen JV, Frisch M, Melbye M (2019). Measles, mumps, rubella vaccination and autism: A nationwide cohort study. Ann Intern Med.

[56] Jansen VA, Stollenwerk N, Jensen HJ, Ramsay ME, Edmunds WJ, Rhodes CJ (2003). Measles outbreaks in a population with declining vaccine uptake. Science.

[57] Jasanoff S (2013). Science and public reason.

[58] Jegou B (2015). Paracetamol-induced endocrine disruption in human fetal testes. Nat Rev Endocrinol.

[59] Kass NE (2001). An ethics framework for public health. Am J Public Health.

[60] Klein RM, Motomura VN, Debiasi JD, Moreira EG (2023). Gestational paracetamol exposure induces core behaviors of neurodevelopmental disorders in infant rats and modifies response to a cannabinoid agonist in females. Neurotoxicol Teratol.

[61] Kocherlakota P (2014). Neonatal abstinence syndrome. Pediatrics.

[62] Kundakovic M, Champagne FA (2015). Early-life experience, epigenetics, and the developing brain. Neuropsychopharmacology.

[63] Larson HJ, Cooper LZ, Eskola J, Katz SL, Ratzan S (2011). Addressing the vaccine confidence gap. Lancet.

[64] Lavery P (2025). Acetaminophen and autism: Industry impacts of the White House Statement. https://www.pharmtech.com/view/acetaminophen-and-autism-industry-impacts-of-the-white-house-statement.

[65] Leask J, Hooker C, King C (2010). Media coverage of health issues and how to work more effectively with journalists: a qualitative study. BMC Public Health.

[66] Leask JA, Chapman S (1998). An attempt to swindle nature: press anti-immunisation reportage 1993-1997. Aust N Z J Public Health.

[67] Legare F, Ratte S, Gravel K, Graham ID (2008). Barriers and facilitators to implementing shared decision-making in clinical practice: update of a systematic review of health professionals' perceptions. Patient Educ Couns.

[68] Leverrier-Penna S, Michel A, Lecante LL, Costet N, Suglia A, Desdoits-Lethimonier C (2021). Exposure of human fetal kidneys to mild analgesics interferes with early nephrogenesis. FASEB J.

[69] Liew Z, Ernst A (2021). Intrauterine exposure to acetaminophen and adverse developmental outcomes: epidemiological findings and methodological issues. Curr Environ Health Rep.

[70] Liew Z, Ritz B, Rebordosa C, Lee PC, Olsen J (2014). Acetaminophen use during pregnancy, behavioral problems, and hyperkinetic disorders. JAMA Pediatr.

[71] Liew Z, Ritz B, Virk J, Olsen J (2016). Maternal use of acetaminophen during pregnancy and risk of autism spectrum disorders in childhood: A Danish national birth cohort study. Autism Res.

[72] Lindsay P (2021). Books: long read: The doctor who fooled the world. Andrew Wakefield's war on vaccines: I looked on immunisation as an example of modern living and progress... and then came Andrew Wakefield. Br J Gen Pract.

[73] Louwen F, Deuster E, McAuliffe FM, Jacobsson B, Geary M, Fleischman S (2025). Paracetamol (acetaminophen) use during pregnancy and autism risk: Evidence does not support causal association. Int J Gynaecol Obstet.

[74] Luxey X, Lemoine A, Dewinter G, Joshi GP, Le Ray C, Raeder J (2025). Acute pain management after vaginal delivery with perineal tears or episiotomy. Reg Anesth Pain Med.

[75] Madsen KM, Hviid A, Vestergaard M, Schendel D, Wohlfahrt J, Thorsen P (2002). A population-based study of measles, mumps, and rubella vaccination and autism. N Engl J Med.

[76] Mazaleuskaya LL, Sangkuhl K, Thorn CF, FitzGerald GA, Altman RB, Klein TE (2015). PharmGKB summary: pathways of acetaminophen metabolism at the therapeutic versus toxic doses. Pharmacogenet Genomics.

[77] Merchant RM, Asch DA (2018). Protecting the value of medical science in the age of social media and "fake news". JAMA.

[78] Morrison A, Polisena J, Husereau D, Moulton K, Clark M, Fiander M (2012). The effect of English-language restriction on systematic review-based meta-analyses: a systematic review of empirical studies. Int J Technol Assess Health Care.

[79] Nappi RE, Tiranini L, Sacco S, De Matteis E, De Icco R, Tassorelli C (2022). Role of estrogens in menstrual migraine. Cells.

[80] Negro A, Delaruelle Z, Ivanova TA, Khan S, Ornello R, Raffaelli B (2017). Headache and pregnancy: a systematic review. J Headache Pain.

[81] Nilsen K, Staff AC, Krogsrud SK (2023). Paracetamol use in pregnancy: Not as safe as we may think?. Acta Obstet Gynecol Scand.

[82] Okubo Y, Hayakawa I, Sugitate R, Nariai H (2026). Maternal Acetaminophen Use and Offspring's Neurodevelopmental Outcome: A Nationwide Birth Cohort Study. Paediatr Perinat Epidemiol.

[83] O'Sullivan J, Cairns AE, Plesca E, Black RS, Frise C, Vatish M (2022). Paracetamol use in pregnancy - neglecting context promotes misinterpretation. Nat Rev Endocrinol.

[84] Pearson H, Ledford H (2025). Trump links autism and Tylenol: is there any truth to it?. Nature.

[85] Petticrew M, Roberts H (2008). Systematic reviews in the social sciences: A practical guide.

[86] Philippot G, Hallgren S, Gordh T, Fredriksson A, Fredriksson R, Viberg H (2018). A cannabinoid receptor type 1 (CB1R) agonist enhances the developmental neurotoxicity of acetaminophen (Paracetamol). Toxicol Sci.

[87] Polifka JE, Friedman JM (2002). Medical genetics: 1. Clinical teratology in the age of genomics. CMAJ.

[88] Prada D, Ritz B, Bauer AZ, Baccarelli AA (2025). Evaluation of the evidence on acetaminophen use and neurodevelopmental disorders using the Navigation Guide methodology. Environ Health.

[89] Raciti M, Ceccatelli S (2018). Epigenetic mechanisms in developmental neurotoxicity. Neurotoxicol Teratol.

[90] Ramos ML (2025). What parents need to know about Tylenol, autism and the difference between finding a link and finding a cause in scientific research. https://theconversation.com/what-parents-need-to-know-about-tylenol-autism-and-the-difference-between-finding-a-link-and-finding-a-cause-in-scientific-research-265946.

[91] Rice D, Barone S (2000). Critical periods of vulnerability for the developing nervous system: evidence from humans and animal models. Environ Health Perspect.

[92] Sackett DL (1998). Evidence-based medicine. Spine (Phila Pa 1976).

[93] Salmon DA, Teret SP, MacIntyre CR, Salisbury D, Burgess MA, Halsey NA (2006). Compulsory vaccination and conscientious or philosophical exemptions: past, present, and future. Lancet.

[94] Sarzi-Puttini P, Giorgi V, Sirotti S, Bazzichi L, Lucini D, Di Lascio S (2024). Pharmacotherapeutic advances in fibromyalgia: what's new on the horizon?. Expert Opin Pharmacother.

[95] Schünemann HJ, Cuello C, Akl EA, Mustafa RA, Meerpohl JJ, Thayer K (2019). GRADE guidelines: 18. How ROBINS-I and other tools to assess risk of bias in nonrandomized studies should be used to rate the certainty of a body of evidence. J Clin Epidemiol.

[96] Sjölander A, Zetterqvist J (2017). Confounders, mediators, or colliders: What types of shared covariates does a sibling comparison design control for?. Epidemiology.

[97] Slovic P (1999). Trust, emotion, sex, politics, and science: surveying the risk-assessment battlefield. Risk Anal.

[98] Szubert M, Stojko R, Sieroszewski P (2025). Statement of the Polish society of gynecologists and obstetricians on paracetamol use during pregnancy and autism risk. Ginekol Pol.

[99] Tadokoro-Cuccaro R, Fisher BG, Thankamony A, Ong KK, Hughes IA (2022). Maternal paracetamol intake during pregnancy-impacts on offspring reproductive development. Front Toxicol.

[100] Talge NM (2020). Prenatal acetaminophen exposure and neurodevelopment: State of the evidence. Paediatr Perinat Epidemiol.

[101] Taylor LE, Swerdfeger AL, Eslick GD (2014). Vaccines are not associated with autism: an evidence-based meta-analysis of case-control and cohort studies. Vaccine.

[102] Tyrrell J, Huikari V, Christie JT, Cavadino A, Bakker R, Brion MJ (2012). Genetic variation in the 15q25 nicotinic acetylcholine receptor gene cluster (CHRNA5-CHRNA3-CHRNB4) interacts with maternal self-reported smoking status during pregnancy to influence birth weight. Hum Mol Genet.

[103] UKTIS, UK Teratology Information Service (2025). Joint Official Position Statement from the UK Teratology Information Service (UKTIS), the MacDonald Obstetric Medicine Society (MOMS), the British Maternal and Fetal Medicine Society (BMFMS) and the Royal College of Obstetricians and Gynaecologists (RCOG): Paracetamol in pregnancy and autism spectrum disorder. https://uktis.org/wp-content/uploads/2025/09/UKTIS-MOMS-BMFMS-Statement-Paracetamol-and-ASD-joint-v2.pdf.

[104] VanderWeele TJ, Ding P (2017). Sensitivity analysis in observational research: Introducing the E-value. Ann Intern Med.

[105] Wakefield AJ, Murch SH, Anthony A, Linnell J, Casson DM, Malik M (1998). Ileal-lymphoid-nodular hyperplasia, non-specific colitis, and pervasive developmental disorder in children. Lancet.

[106] Werler MM, Mitchell AA, Hernandez-Diaz S, Honein MA (2005). Use of over-the-counter medications during pregnancy. Am J Obstet Gynecol.

[107] Wisner K (2021). Partnering with patients and families to prevent maternal morbidity and mortality. MCN Am J Matern Child Nurs.

[108] Wu K, Lu W, Yan X (2023). Potential adverse actions of prenatal exposure of acetaminophen to offspring. Front Pharmacol.

[109] Yoon E, Babar A, Choudhary M, Kutner M, Pyrsopoulos N (2016). Acetaminophen-induced hepatotoxicity: a comprehensive update. J Clin Transl Hepatol.

[110] Ystrom E, Gustavson K, Brandlistuen RE, Knudsen GP, Magnus P, Susser E (2017). Prenatal exposure to acetaminophen and risk of ADHD. Pediatrics.

